# Who initiates price competition when generic entrants are introduced into the South Korean pharmaceutical market?

**DOI:** 10.3389/fpubh.2022.934161

**Published:** 2022-09-14

**Authors:** Kyung-Bok Son

**Affiliations:** College of Pharmacy, Hanyang University, Ansan, South Korea

**Keywords:** competition, pharmaceutical, generic entrants, multilevel modeling, South Korea

## Abstract

**Background:**

Price competition has the potential to reduce health expenditures without hindering pharmaceutical innovation. However, empirical evidence on price competition after generic drugs are introduced is scarce. This study investigates product- and substance-level determinants of price competition following the entry of generics into the South Korean market.

**Methods:**

We selected substances that were approved by the Ministry of Food and Drug Safety from 2000 to 2019, linked their corresponding pharmaceutical products, measured the degree of price competition under various scenarios, and utilized multilevel analysis to investigate the determinants of price competition.

**Results:**

A total of 986 substances and 12,109 corresponding pharmaceutical products were identified. Only 11% of products were affected by price competition in the 10% scenario. However, the number increased to 43% when we measured price competition at the substance level. Major domestic manufacturers mainly initiated price competition at the product level, while foreign manufacturers were reluctant to initiate price competition. At the substance level, the maximum reimbursement price was a significant determinant of price competition.

**Conclusion:**

Price competition at the product level is rare in South Korea. In contrast, the market is quite price competitive at the substance level. Policy options could be introduced to encourage “discounted generic” substitution in an effort to maximize the effects of price competition at the substance level. Major domestic manufacturers are essential in the introduction of discounted generics into the South Korean health system.

## Background

The term “generics” is commonly applied to consumer products having no brand name or registered trademark. In the context of the pharmaceutical market, generic drugs contain the same active substance as reference drugs (1, 2) and are marketed after the patent or statutory exclusivity period of the reference drug has expired. Generics are authorized to be perfect substitutes for the reference drug (3, 4), and in fact, bioavailability tests are required before authorities will grant marketing approval for generic drugs (5). The development costs to manufacture generic drugs are less expensive than the costs associated with introducing originators into the market. The price competition resulting from the entry of generics into the market has the potential to reduce health expenditures without hindering pharmaceutical innovation (6, 7).

Many countries have adopted price regulations for originators and generic drugs entering the market (8–11). When a generic enters the European market, the price of the originator falls by an average of 20% during the first year after the loss of exclusivity, with an additional 5% loss over the next two years (8). Due to the abbreviated nature of required testing in generic drugs, generics are 20–80% less expensive than originators (8, 12–14). Manufacturers of generic drugs engage in price competition and successfully penetrate originator markets in many high-income countries (15–17). In a recent study, the actual price charged by generic manufacturers in the United Kingdom fell by 70% in the 6 months after the loss of exclusivity, with an additional 10–20% loss over the next 4 years (18).

South Korea has a unique National Health Insurance (NHI) program, and 97% of the Korean population is covered under the program (19). Prescription drugs are included in the NHI benefit packages. As in other countries, the prices of originators and generic drugs are regulated. When markets set strict price regulations on originators and generic drugs after generic entrance, prices cluster around the maximum reimbursement price (10, 11). However, the number of generic drugs in South Korea is greater than the numbers of generic drugs in the United States, the United Kingdom, Germany, and Japan. For instance, in South Korea, there are more than 100 generic versions of well-known blockbuster drugs (20, 21). Generic competition is linked to lower drug prices in other markets (22–24). It is reasonable to expect price competition among generic drugs. Nevertheless, the high number of generic drugs has not triggered price competition in the South Korean market (20).

Numerous pharmaceutical products including the same active substances are available in South Korea (20, 21). Positive associations between price variance of active substances and the number of generic drugs including those substances were recently reported (24). Previous research on the topic is limited by its narrow focus on substance characteristics without considering product characteristics (24). In this study, we investigate product- and substance-level determinants of price competition following the entry of generics into the South Korean market. To this end, we took advantage of a data structure to conduct a multilevel analysis in which products (level one) were nested within substances (level two). The study design compelled us to deal with characteristics at level one and at level two simultaneously.

## Methods

This study explores price competition among pharmaceutical products following the market entry of generic drugs into the South Korean market. We selected substances that were first approved by the Ministry of Food and Drug Safety (MFDS) between 2000 and 2019, linked the corresponding products with the same generic and proprietary name, strength, and route of administration, and conducted a multilevel analysis to investigate product- and substance-level determinants of price competition.

### Data sources

We used three data sources for the study. First, all reimbursed pharmaceutical products in the National Health Insurance (NHI) database were retrieved as of June 2020. The database, which was provided by the Health Insurance Review and Assessment Service (HIRA), includes information on each product and its manufacturer. The generic and proprietary name, strength, route of administration, and unit price of each product were obtained from the database. Second, all eligible products were identified through the Korea Pharmaceutical Information Service (KPIS) website (25), which provides information on each product, manufacturer, and date of marketing authorization. Finally, KISVALUE (26), an analytic dataset that was created to elucidate manufacturers' financial resources, was used to capture manufacturer characteristics.

### Variables

#### Dependent variables

This study examines price competition among originators and generics following the market entry of generics. The South Korean government sets the maximum reimbursement price of originator and generics after the market entry of generics, and manufacturers can voluntarily discount their price to be lower than the aforementioned maximum reimbursement price (27, 28). The price of originators falls by about 30% during the first year of loss of exclusivity (27, 28). The maximum reimbursement price of generic drugs is set at 59.5% of the price of the corresponding originator prior to loss of exclusivity. After 1 year, the maximum price of both the originator and generic drugs is set at 53.55% of the originator's price before the loss of exclusivity (27, 28). We retrieved prices of pharmaceutical products and maximum reimbursement prices of substances from the HIRA in June 2020. Using this publicly available information, we measured the price discount of each product and then identified price competition. We calculated the price discount at the product level as the difference between the maximum reimbursement price of the substance and the price of the investigated product divided by the maximum reimbursement price. The mean price variance among pharmaceutical products nested in the same substance was 11.67% (24). Given this value, we defined price competition at the product level as a binary variable and coded it as 1 if the product discount was more than 10%.

#### Variables of interest

The variables of interest at level one were the characteristics of manufacturers and drug lag. Using the KISVALUE dataset, manufacturers were categorized into three groups: foreign manufacturers, major domestic manufacturers, and medium- or small-sized domestic manufacturers. Drug lag was defined as the difference in years between the date of marketing approval of the first pharmaceutical product containing a given substance and the product containing the same substance. The KPIS dataset includes the dates of marketing authorization of eligible products. For variables of interest at level two, we used the year of marketing approval for the first product containing the substance, the maximum reimbursement price of the substance, and the number of products containing the substance. Given its right skewed distribution, maximum reimbursement price was log transformed before use. Numbers of products sharing active substances were categorized into four groups: duopoly (2 products), 3–25 products, 26–75 products, and more than 75 products.

### Model specification

We took advantage of a data structure in which pharmaceutical products (level one) were nested within substances (level two). In previous studies, we found that the characteristics of a substance were associated with the price variance of the substance (24). We hypothesized that the characteristics of pharmaceutical products would be associated with price competition. We used a two-level hierarchical model to consider product-related variables at level one and substance-related variables at level two simultaneously. The multilevel model represents price competition for pharmaceutical product *i* within substance *j*. At level one, a separate product-level logistic regression model was defined for each substance. At level two, level one coefficients were modeled as functions of level two variables. The term γ_00_ indicates the average log-odds of price competition across substances, whereas u_0j_ presents substance-specific intercepts. Numeric variables were group-centered at level one and were grand-mean centered at level two. Categorical variables were not treated as centered.

***Level one model***


$$\begin{array}{l} logit_{ij}=log\left[Prob\left(PC_{ij}\right)/\left(1-Prob\left(PC_{ij}\right)\right)\right]\\ =\beta_{0j}+\beta_{1j}^{*}\left(x1_{ij}\right)+\beta_{2j}^{*}\left(x2_{ij}\right) \end{array}$$

***Level two model***


$$\begin{array}{l} \beta_{0j}=\gamma_{00}+\gamma_{01}^{*}\left(a1_{j}\right)+\gamma_{02}^{*}\left(a2_{j}\right)+\gamma_{03}^{*}\left(a3_{j}\right)+u_{0j}\\ \beta_{1j}=\gamma_{10}+\gamma_{11}^{*}\left(a1_{j}\right)+\gamma_{12}^{*}\left(a2_{j}\right)+\gamma_{13}^{*}\left(a3_{j}\right)+u_{1j}\\ \beta_{2j}=\gamma_{20}+\gamma_{21}^{*}\left(a1_{j}\right)+\gamma_{22}^{*}\left(a2_{j}\right)+\gamma_{23}^{*}\left(a3_{j}\right)+u_{2j} \end{array}$$

*PC: price competition*.

*x1 and x2 are level one predictors*.

*a1, a2, and a3 are level two predictors*.

We applied a null model (model 0), a model with level one variables (model 1), a model with level one and level two variables (model 2), and a model with interaction effects between variables at level one and level two (model 3) to predict price competition. The null model was estimated to investigate whether differences in price competition could be found at the product level or at the substance level. Chi-square tests were used to investigate whether each model fitted the data significantly better than the null model. In a similar vein, we used AIC and BIC to check the goodness of fit of the models. Data management and analysis were performed using R statistical software (version 4.1.3). Particularly, the “lme4” and “lmerTest” package in R statistical software was used to conduct a multilevel logistic regression. Statistical significance was defined as a *p* < 0.05.

### Sensitivity analysis

The mean price variance among pharmaceutical products nested in the same substance was 11.67% (24). Given this value, price competition was defined as a discount of >10% in the main analysis. We defined price competition in various ways, including as a discount of >7% and >15%, for sensitivity analyses.

## Results

### Descriptive statistics

Our analysis included 986 substances that were first granted marketing approval between 2000 and 2019. A total of 12,109 pharmaceutical products were identified for the analysis. Table 1 summarizes the descriptive statistics of all variables included in the model. At level one, the products were grouped into price competition and non-competition categories. Price competition was defined in various ways, such as 7, 10, and 15% discount scenarios. Based on the 10% scenario, 1,381 (11%) out of 12,109 products showed evidence of price competition. The proportion of products exhibiting price competition decreased as the reference point increased. For instance, 14 and 7% of products presented price competition in the 7 and 15% scenarios, respectively. In all scenarios, the types of manufacturers and drug lag measured in years were significantly different between the price competition and non-competition groups. In the 10% scenario, 850 (16.5%) out of 5,154 products produced by major domestic manufacturers exhibited price competition. In comparison, 7.7 and 6.9% of products produced by small- or medium-sized domestic manufacturers and foreign manufacturers, respectively, exhibited price competition. In the same scenario, the average drug lag was shorter for products with price competition (6.59 years) than for those without price competition (7.22 years).

**Table 1:** Descriptive statistics of variables at level one and level two.

**Scenarios**	**7%**			**10%**			**15%**		
	**Non-**	**Price**	***P*-value**	**Non-**	**Price**	***P*-value**	**Non-**	**Price**	***P*-value**
	**competition**	**competition**		**competition**	**competition**		**competition**	**competition**	
**Level one (12,109 products)**	(*n* = 10,370)	(*n* = 1,739)		(*n* = 10,728)	(*n* = 1,381)		(*n* = 11,212)	(*n* = 897)	
Types of manufacturers			<0.0001			<0.0001			<0.0001
Foreign	466	43		474	35		491	18	
Major domestic	4,056	1,098		4,304	850		4,625	529	
Medium- or small-sized domestic	5,848	598		5,950	496		6,096	350	
Drug lag	7.21 (5.43)	6.44 (4.67)	<0.0001	7.23 (5.41)	6.59 (4.73)	<0.0001	7.21 (5.38)	6.44 (4.78)	<0.0001
**Level two (986 substances)**	(*n* = 489)	(*n* = 497)		(*n* = 559)	(*n* = 427)		(*n* = 678)	(*n* = 308)	
Year of first marketing approval	2,008 (5.81)	2,006 (4.69)	<0.0001	2,008 (5.77)	2,006 (4.55)	<0.0001	2,008 (5.63)	2006 (4.47)	<0.0001
Maximum reimbursement price	7.32 (2.27)	7.63 (2.14)	0.0296	7.34 (2.27)	7.65 (2.12)	0.0288	7.43 (2.26)	7.57 (2.10)	0.3647
Number of products			<0.0001			<0.0001			<0.0001
2 (duopoly)	199	106		215	90		244	61	
3–25	265	289		306	248		390	164	
26–75	21	67		30	58		33	55	
>76	4	35		8	31		11	28	

At level two, any substance that included at least one product with price competition was counted in the price competition group. Approximately 50, 43, and 31% of substances were defined as price-competitive in the 7, 10, and 15% scenarios, respectively. We summarized the year of first marketing approval, the maximum reimbursement price, and the number of manufacturers among the price competition and non-competition groups. In all scenarios, the year of marketing authorization and the number of manufacturers differed significantly between the two groups. In the 10% scenario, the mean year of first marketing approval was 2008 and 2006 for the non-competition and price competition groups, respectively. The maximum reimbursement price after log transformation was higher for products in the price competition group than in the non-competition group in the 7 and 10% scenarios. The maximum reimbursement price, however, was not significantly different between the two groups in the 15% scenarios.

### Multilevel modeling

Table 2 presents odds ratios for price competition (more than 10% discount) in the market. At level one, manufacturers' characteristics were associated with price competition in models 1, 2, and 3. The odds of price competition for products produced by major domestic manufacturers were greater than the odds of price competition for products produced by foreign manufacturers in model 3B (adjusted odds ratio (AOR) = 10.58, *p* < 0.0001). In the same model, the odds of price competition for products produced by small- or medium-sized domestic manufacturers were greater than the odds of price competition for products produced by foreign manufacturers (AOR = 5.01, *p* < 0.0001). However, drug lag was not a consistent predictor in models 1, 2, and 3. Drug lag was associated with decreased odds of price competition in model 1 (AOR = 0.95, *p* = 0.0251). However, the variable was not a significant predictor in models 2A, 2C, 3A, and 3B.

**Table 2:** Estimated odds ratios for price competition with discount of more than 10%.

	**Model 0**	**Model 1**	**Model 2A**	**Model 2B**	**Model 2C**	**Model 3A**	**Model 3B**
**Fixed effects**							
**A. Level one (products level)**							
Manufacturers (Ref foreign)							
Major		9.69***	9.73***	9.98***	11.19***	10.88***	10.58***
Medium- or small-sized		4.53***	4.56***	4.62***	5.21***	5.02***	5.01***
Drug lag		0.95*	0.95	0.95*	0.96	1.03	0.96
**B. Level two (substance level)**							
Number of products (Ref. 2)							
3–25			1.05	0.95	1.05	1.04	1.08
26–75			0.97	0.83	0.96	0.86	0.98
>76			0.56	0.43*	0.53	0.47	0.53
Year of first marketing approval				0.92***	0.93***	0.93***	0.93***
MRP					1.24***	1.24***	1.27***
**C. Interaction**							
Drug lag X year of marketing approval						1.02***	
Drug lag X MRP							1.05***
**Random effects**							
Random intercept	2.99	4.46	4.18	4.03	3.63	3.45	3.70
Random slope		0.06	0.06	0.05	0.05	0.04	0.04
Covariance		0.25	0.21	0.20	0.14	0.08	0.14
**Goodness of fit**							
AIC	7,180.6	6,715.2	6,718.4	6,700.0	6,675.9	6,668.0	6,647.7
BIC	7,195.4	6,767.0	6,792.4	6,781.4	6,764.7	6,764.2	6,744.0
χ^2^		475.41	478.18	498.59	524.67	534.59	554.84
df		5	8	9	10	11	11
P		<0.0001	<0.0001	<0.0001	<0.0001	<0.0001	<0.0001
reference		Model 0	Model 0	Model 0	Model 0	Model 0	Model 0

*p < 0.05, **p < 0.001, ***p < 0.0001.

At level two, the year of marketing approval, the maximum reimbursement price was a consistent predictor in models 2 and 3. However, the variables had different effects on price competition. In model 3B, increases in the year of first marketing approval (AOR = 0.93, *p* < 0.0001) were associated with decreased odds of price competition, while an increase in the maximum reimbursement price increased the odds of price competition (AOR = 1.27, *p* < 0.0001). We found interactions between the variables of drug lag at level one and maximum reimbursement price at level two in model 3B. The maximum reimbursement price was a significant predictor of price competition (AOR = 1.27, *p* < 0.0001), and the effects were stronger when drug lag increased (AOR = 1.05, *p* < 0.0001).

### Sensitivity analysis

Table 3 summarizes the findings of sensitivity analysis under various scenarios. Given its goodness of fit, we chose model 3B for sensitivity analysis. We used three different discount scenarios (7, 10, and 15% discounts), and found that some results were consistent across all scenarios. The characteristics of manufacturers at level one, the year of marketing approval and maximum reimbursement price at level two, and the interaction between drug lag and maximum reimbursement price were robust in all scenarios.

**Table 3:** Estimated odds ratios for price competition in various scenarios.

**Scenarios**	**7%**		**10%**		**15%**	
	**Odds ratio**	***P*-Value**	**Odds ratio**	***P*-Value**	**Odds ratio**	***P*-Value**
**A. Level one (products level)**
Manufacturers (Ref foreign)						
Major	15.11	<0.0001	10.58	<0.0001	12.44	<0.0001
Medium- or small-sized	6.64	<0.0001	5.01	<0.0001	7.59	<0.0001
Drug lag	0.95	0.0062	0.96	0.1080	0.90	0.0005
**B. Level two (substance level)**
Number of products						
3–25	1.27	0.2523	1.08	0.7290	0.75	0.2840
26–75	1.01	0.9641	0.98	0.9550	1.27	0.5479
>76	0.67	0.2944	0.53	0.1220	0.62	0.3291
Year of first marketing approval	0.93	<0.0001	0.93	0.0001	0.93	0.0009
MRP	1.30	<0.0001	1.27	<0.0001	1.26	<0.0001
**C. Interaction**
Drug lag x MRP	1.04	<0.0001	1.05	<0.0001	1.05	<0.0001

MRP, maximum reimbursement price.

## Discussion

Empirical evidence regarding price competition among the large numbers of generic drugs available in South Korea is scarce. Price competition among pharmaceutical products with the same active substance compelled us to address characteristics of products and substances simultaneously. Our study provides up-to-date evidence on price competition by utilizing a multilevel modeling technique. Our findings have the potential to provide insights for policy measures to manage pharmaceutical expenditures through price competition among generic drugs.

First, we confirmed that price competition at the product level is rare in South Korea. Based on the 10% scenario, only 1,381 (11%) out of 12,109 products were shown to be price-competitive. When measuring price competition at the substance level, however, 427 (43%) out of 986 substances showed price competition. Second, we found that major domestic manufacturers typically initiate price competition at the product level. Foreign manufacturers were more reluctant than major domestic manufacturers to initiate price competition. Medium- or small-sized domestic manufacturers had moderate tendencies toward initiating price competition. Third, we observed a counterintuitive effect of drug lag on price competition. In principle, the manufacturers that are granted later marketing approval (“latecomers”) would be expected to proactively drop their prices in an attempt to penetrate markets that have already matured (22–24, 29). In fact, our models show that drug lag was not a significant predictor of price competition.

### Variation in price competition at two levels

In this study, we measured price competition at two levels. At level one, only 11% of pharmaceutical products exhibited price competition in the 10% scenario. In contrast, this value increased to 43% in the same scenario when we applied price competition at level two. Note that any substance that included at least one product with price competition was counted in the price competition group. This finding is consistent with the findings of previous studies (20, 24). One study showed that 75% (82 out of 109) of choline alfoscerate drugs in capsule form retained the maximum reimbursement price of the substance (20). In contrast, the mean price variance among pharmaceutical products containing the same substance has been reported to be 11.67% (24).

Some noteworthy variations in price competition were measured at two levels and suggested that “discounted generic” substitution policies could take advantage of price competition at the substance level. However, the scale of the generic market is relatively small in South Korea. In 2018, the retail market share of generic drugs was 55% in South Korea, making it much smaller than the retail market share of generic drugs in other OECD countries (30). The market shares of generic drugs in the United States (81%), Germany (77%), Canada (76%), and the United Kingdom (71%) all exceeded 70% in the same year (30). In contrast, the average penetration of generics in certain markets in South Korea was reported to be 26.63% in one study (31) and 39.35% in another (32). Policy options that enhance “discounted generic” substitution could be broadened or strengthened to maximize the effects of price competition at the substance level (1, 33, 34).

### Role of major domestic manufacturers in health systems

Previous studies described the accumulation of generic drugs in the South Korean market. Notably, Son (21, 24) categorized generic drug manufacturers as either first-movers or latecomers (21). The designation of first-movers refers to manufacturers entering the market within 2.5 years of the date of the first generic entrant, while latecomers describes manufacturers entering the market after 2.5 years. Latecomers continue to enter mature markets that have been populated by many first-movers. In this way, latecomers ultimately account for the largest portion of the generic drug market. The sizes of manufacturers based on their financial resources are closely associated with their status as a first-movers or latecomers. Major domestic manufacturers have been shown to be more likely to be first-movers.

Compared to foreign manufacturers, major and small- or medium-sized domestic manufacturers were more aggressive at initiating price competition. In particular, major domestic manufacturers play an essential role in health systems insofar as they introduce discounted generics. In this study we also found that foreign manufacturers were more likely to retain the maximum reimbursement price than major domestic manufacturers. In South Korea, foreign manufacturers mainly introduce brand-name drugs into the market, and can thus expect brand loyalty from physicians or patients (35). Brand loyalty implies that foreign manufacturers are reluctant to compete on price with generic entrants, which is a common phenomenon in the United States and Canada (36–38).

### Order-of-entry effects in South Korea

When it comes to manufacturers entering the market, order of entry affects the market share of products. This order-of-entry effect assumes that the market favors early entrants due to the benefits of preemption and the brand loyalty of customers (39). Order-of-entry effects have been empirically demonstrated in various pharmaceutical markets (40, 41). Late entrants to a market cannot anticipate high market share for their products. Accordingly, they initiate price competition to overcome their weaknesses in the market and to penetrate a market that consists mainly of early entrants (22–24, 29). Aggressive price competition among late entrants is partially associated with the limited number of generic entrants in other markets. Late entrants are reluctant to enter markets or more willing to offer their products at a discounted price. We observed counterintuitive phenomena in South Korea. First, many late entrants chose to enter the market even after the market had matured. In the sample herein, for instance, 127 out of 986 substances (12.8%) were offered by more than 25 manufacturers. Surprisingly, 39 substances (4.0%) were produced by more than 75 manufacturers. Second, drug lag was not a significant factor predicting price competition in our multilevel analysis. Finally, products with increased drug lag were associated with decreased odds of price competition in some scenarios.

How can we explain such counterintuitive phenomena in the South Korean market? Manufacturers anticipate profit when they decide to enter the market. Profit is calculated by deducting total costs, such as material and labor costs and overhead, from total revenue. In the pharmaceutical market, total revenue is determined by unit price and sales volume. In markets that include aggressive price competition among generic entrants, manufacturers mainly anticipate profits from increased sales. However, manufacturers might also anticipate profits from a product with a high unit price if late entrants have not initiated aggressive price competition. In particular, strict price regulation on generics would result in prices clustering around the maximum reimbursement price with little competition (10, 11). Lack of price competition among generic entrants in South Korea is well-documented in the existing literature (21, 42). Indeed, South Korean manufacturers can reasonably anticipate profits due to higher unit prices, even in cases of late market entry.

### Limitations

This study has several limitations. First, we analyzed price competition among pharmaceutical products following the entry of generics into the South Korean markets. Single-source off-patent or off-exclusivity pharmaceutical products were not included in this study. Second, the lack of availability of certain data should be considered when interpreting the results. We were unable to control for the market size of each substance and the market share of each product in the multilevel model. Including these variables in future research may be beneficial for better understanding. Third, we analyzed pharmaceutical price competition in South Korea, where the prevalence of a large number of generic entrants and a lack of price competition have been reported. The findings of this study may not be generalizable to other markets where a limited number of manufacturers with aggressive price competition are the norm.

## Conclusion

Price competition among pharmaceutical products is rare in South Korea. In our analysis, only 11% of products were shown to be price-competitive in the 10% scenario. However, this value increased to 43% when we measured price competition at the substance level. Policy options could be introduced to encourage “discounted generic” substitution in order to maximize the effects of price competition at the substance level. Major manufacturers are essential to health systems insofar as they introduce discounted generics and initiate price competition more actively than other types of manufacturers.

## Data availability statement

The study analyzed publicly accessible datasets (https://www.hira.or.kr/bbsDummy.do?pgmid=HIRAA030014050000; https://biz.kpis.or.kr/kpis_biz/index.jsp?sso=ok).

## Author contributions

K-BS designed the study, collected and analyzed data, and wrote the manuscript.

## Funding

This work was supported by the research fund of Hanyang University (HY-2022-0982).

## Conflict of interest

The author declares that the research was conducted in the absence of any commercial or financial relationships that could be construed as a potential conflict of interest.

## Publisher's note

All claims expressed in this article are solely those of the authors and do not necessarily represent those of their affiliated organizations, or those of the publisher, the editors and the reviewers. Any product that may be evaluated in this article, or claim that may be made by its manufacturer, is not guaranteed or endorsed by the publisher.
